# Weight-for-age and mid-upper arm circumference versus weight-for-length as severe acute malnutrition indicators: A validation study in under-6-month infants

**DOI:** 10.6026/973206300222305

**Published:** 2026-04-30

**Authors:** Kumari Pinky, Rashmi Randa Mahore, Shiva Saloni Thakur, Sachin Parmar

**Affiliations:** 1 Department of Paediatrics, Government Medical College, Satna, Madhya Pradesh, India; 2 Department of Pediatrics, GMC Bhopal, Madhya Pradesh, India; 3 Department of Pediatrics, Civil hospital Amarwara, Chhindwara, Madhya Pradesh, India; 4 Department of Community Medicine, V.K.S. Government Medical College, Neemuch, India

**Keywords:** Severe acute malnutrition (SAM), mid-upper arm circumference (MUAC), infants under six months

## Abstract

Infants are more susceptible to malnutrition than older children. The prevalence of severe wasting among Indian infants under-6 months of age is 14.8% according to the National Family Health Survey-4. The myth of complete protection by exclusive breastfeeding is debated when the prevalence of severe acute malnutrition (SAM) sprang up as high as 23.8% in a cross sectional study from Central India among 600 infants. Area under the curve for MUAC <11 cm was 0.936 (95% confidence interval); its sensitivity and specificity were 69.23% and 73.09%, respectively; area under the curve for weight-for-age had higher sensitivity at (88.81%), but lower specificity (61.1%). The highlighted modifiable risk factors were low birth weight and adverse feeding practices. Combined MUAC and weight-for-length screening strategies should be used specifically in low-resource, community-based contexts for SAM in infants aged under six months.

## Background:

An estimated 8.5 million infants less than 6 months are wasted globally [1]. These infants have much higher rates of morbidity and mortality compared to older malnourished children [2]. In India, the burden of under-nutrition in this age group is grossly underestimated owing to insufficiency of screening tools [3, 4]. Infants are more vulnerable to malnutrition as compared with older children [5]. The World Health Organization (2013) recommends using weight-for-length z-score (WLZ), rather than WHZ, as the principal indicator for the identification of acute malnutrition in infants below six months [6]. But that recommendation is made on very low-quality evidence from extrapolate conventions for older age groups [7]. The WHO growth standards do not cover infants shorter than 45 cm; hence it is impossible to calculate the WLZ of the most vulnerable ones [8]. Measurement of length in young infants is also problematic because of hip and knee flexed positions, infant movement and the lack of experience from handlers [9] to compound this issue, WLZ combines two measurements together and will, therefore, combine existing errors which makes it the least reliable anthropometric indicator [10]. Mid-upper arm circumference (MUAC) and weight-for-age z-score (WAZ) have shown the most promise as mortality predictors in infants < 6 months [8]. Studies from Kenya showed AUC of 0.77 and 0.76 for MUAC and WAZ compared to only 0.71 for WLZ for mortality in inpatient management, using a cut-off at MUAC <110 cm identified 24% of at-risk infants with sensitivity and specificity ratio of >64%:70% [9]. In the Gambia community cohorts, MUAC was noted to have superior prognostic ability when compared with WLZ, irrespective of age [11].

A systematic review recommended WAZ and/or MUAC over WLZ, with optimal thresholds varying from 10.5-11.5 cm depending on the context [7]. As such, an analysis of low- and middle-income country DHS datasets across 56 countries highlighted the underestimated burden of early infant malnutrition [12, 13]. Programme caseload estimates have a marked implication based on MUAC based thresholds used in application of WHO-2023 criteria [14]. Still, we know that inadequate feeding practices like lack of colostrum can lead to early feeds deficits. Ineffective maternal nutrition and low birth weight are the major modifiable risk factors for SAM among Indian infants [15]. Even infants who are exclusively breastfed can be severely wasted if they were low birth weight or from poorly nourished mothers [16]. This research is one of the first to validate context- specific MUAC and WAZ thresholds for SAM screening in infants less than six months of age in a resource-poor tertiary care setup in central India. MUAC <11.0 cm demonstrated the best-balance sensitivity (69.23%) and specificity (73.09%). It provides regionally relevant anthropometric cut-off values not currently available in national screening guidelines. Therefore, it is important for the SAM screening of infants less than 6 months in a resource-constrained central Indian setting, it is worthwhile to report context specific MUAC and WAZ thresholds.

## Materials and Methods:

## Study design and clinical setting:

This investigation was designed as a hospital-based observational study, conducted at the Department of Pediatrics, Gandhi Medical College and its associated tertiary care hospitals in Bhopal, Madhya Pradesh. This region of Central India is characterized by a diverse demographic profile, serving as a referral hub for both urban and rural populations, which ensures that the study cohort is representative of the broader epidemiological landscape of the region. The study was executed over a period of eighteen months, commencing in March 2021 and concluding in September 2022. This duration was sufficient to capture seasonal variations in morbidity and nutritional status, providing a robust dataset for analysis.

## Study population and sampling strategy:

The study enrolled infants aged between 1 month and 6 months who presented to the Outpatient Department (OPD) of the pediatric clinic. The exclusion of neonates (0-1 month) was deliberate, as anthropometric variability in the immediate neonatal period is heavily influenced by fluid shifts and transitional physiology.

## Inclusion criteria:

All infants aged 1 to 6 months presenting to the OPD during the study period were screened for eligibility.

## Exclusion criteria:

A critical exclusion criterion was a body length of less than 45 cm. This cut off is mandated by the WHO Child Growth Standards, which do not provide weight-for-length standard deviation values for lengths below 45 cm. Consequently, infants shorter than this threshold cannot be assessed using the gold standard WLZ metric, necessitating their exclusion from a comparative validation study.

## Data collection protocols:

Ethical integrity was a cornerstone of the study protocol. Ethical clearance was secured from the Institutional Ethical Committee of Gandhi Medical College, Bhopal. Written informed consent was obtained from the parents or legal guardians of all participants, with the consent forms provided in a bilingual format to ensuring full comprehension of the study's observational nature. A structured proforma was utilized to collect comprehensive demographic and clinical data. This included:

## Demographics:

Age, gender, residential status (urban/rural) and socio-economic status classified according to the Modified Kuppuswamy Scale.

## Clinical history:

Detailed records of birth weight, gestational maturity (term/preterm), history of prior hospitalizations and vaccination status were compiled.

## Dietary history:

Feeding practices were rigorously categorized into exclusive breastfeeding, mixed feeding (breast milk plus other liquids/solids), or exclusive top feeding.

## Anthropometric assessment:

To ensure the internal validity of the study, anthropometric measurements were performed by trained personnel following strict standardized techniques.

## Weight:

Infants were weighed using a calibrated digital weighing scale. To minimize measurement error, all clothing and diapers were removed. The infant was positioned to distribute weight evenly across the pan and measurements were recorded to the nearest 10 grams.

## Length:

Recumbent length was measured using a highly precise infantometer. This procedure required two individuals: an assistant to hold the infant's head in the Frankfurt plane (auditory meatus - lower margin of the orbit line horizontal) against the fixed headboard and the principal investigator to extend the infant's legs, pressing the knees down gently and bringing the movable footboard firmly against the soles of the feet. Measurements were recorded to the nearest 0.1 cm.

## Mid-Upper Arm Circumference (MUAC):

MUAC was measured on the left arm using a flexible, non-stretchable measuring tape. The midpoint between the acromion process (tip of the shoulder) and the olecranon process (tip of the elbow) was identified with the arm flexed at 90 degrees. The arm was then relaxed and extended and the tape was placed around the midpoint perpendicular to the long axis of the arm. Care was taken to ensure the tape was snug against the skin without compressing the underlying soft tissue. Measurements were recorded to the nearest 0.1 cm.

## Head circumference:

This was measured using a non-stretchable tape passed over the supraorbital ridges anteriorly and the maximum occipital protuberance posteriorly.

## Diagnostic definitions:

The nutritional status of each infant was categorized based on the WHO 2006 Child Growth Standards:

## Severe acute malnutrition (SAM):

Defined as a Weight-for-Length Z-score (WLZ) < -3 SD and/or bilateral pitting edema and/or visible severe wasting.

## Moderate acute malnutrition (MAM):

Defined as a WLZ between -2 SD and -3 SD.

## Underweight:

Defined as Weight-for-Age Z-score (WAZ) < -2 SD. Severe underweight was classified as WAZ < -3 SD.

## Stunting:

Defined as Length-for-Age Z-score (LAZ) < -2 SD. Severe stunting was classified as LAZ < -3 SD.

## Results and Discussion:

Among the 600 infants enrolled (61.9% male), the age group of 2-3 months was the most represented (35.1%). The prevalence of SAM was 23.8% and MAM was 12.2% (Table 1 - see PDF). Of all the cut-offs for mid-upper arm circumference (MUAC), <11.5 cm had the maximum sensitivity (86.01%), whereas <10.0 cm had the best diagnostic accuracy (77.5%). Weight-for-Age (<-2 standard deviation) had high sensitivity (88.81%) but overall low accuracy (62.83%), thus limiting standalone use (Table 2, Table 3 - see PDF). Low birth weight (62.80% with SAM vs. 39.30% without SAM, p<0.001), prematurity (p=0.006) and faulty feeding (p=0.024) were all significant risk factors for SAM. Prior hospitalization was not a significant risk factor (p=0.059) (Table 4 - see PDF). Table 5 (see PDF) shows that there was a significantly lower rate of exclusive breastfeeding at SAM cases (65.7%) in comparison to normally nourished infants (75.0%). The SAM cases had mixed feeding at 34.3%, which something that does not occur in normal infants. This indicates that optimal feeding is a protective factor. The demographic study of the sample population indicated a malnutrition burden during the early infancy, which debunks the historical belief that infants below six months (u6m) of infancy are immune to nutritional deficits due to breastfeeding. Among the infants in our cohort, 23.8% were found to have Severe Acute Malnutrition (SAM) and 12.2% had Moderate Acute Malnutrition (MAM) according to the weight-length z-scores (WLZ) (Table 1 - see PDF). This is shockingly large and in line with the new world data; Kerac *et al.* carried a population-weighted prevalence of wasting in infants u6m in 56 low- and middle-income countries found to be 15.5, with an estimated 9.2 million infants being affected worldwide [10]. The results of our study are consistent with the age-prevalence inversion in the case of Patwari *et al.* who found that in a secondary analysis of National Family Health Survey data of India that in infants u6m, wasting was significantly higher than in children aged 6-59 months, which is in contrast with the classic epidemiological curve, which reaches its peak in the weaning age [11]. Interestingly, the sample of 61.9% of the babies in our study were males, which is also usually explained by the fact that male babies are more vulnerable biologically and more studies should be conducted on sex-specific vulnerability. Our study's finding of male predominance is in line with other studies. In a study at a tertiary health institution in Gujarat, India, Bakhalakiya *et al.* [12] reported 61.9% males in children below 5 years and 38.1% females evaluated for severe acute malnutrition. The analysis of diagnostic accuracy revealed the weaknesses associated with the use of WLZ alone as well as the usefulness of Mid-Upper Arm Circumference (MUAC) (Table 2 - see PDF). We found a MUAC cut-off of < 11.0 cm to have a diagnostic accuracy of 72.17% and a specificity of 73.09% and a cut-off of < 11.5 cm to increase sensitivity to 86.01% but decrease specificity to 58.42%. This is comparable to Zehra *et al.* in Pakistan who found that a cut-off of ≤ 11.5 cm in MUAC was giving a sensitivity of 59.5 and specificity of 71.4 thus accepted it as a valid screening tool [13]. But the discussion about the optimum value continues; in a large cohort study in Kenya Mwangome *et al.* have found that MUAC < 11.0 cm was better than WLZ < -3 to predict inpatient mortality, regardless of age [6]. The discrepancy between our results indicators, in which weight-for-age (WAZ) below -2 SD was found to have a sensitivity of 88.81% (Table 3 - see PDF), supports the 2023 World Health Organization (WHO) guideline change. Negesse *et al.* demonstrated the practical implication of these new criteria in Ethiopia to provide that the caseload eligible to treatment is increased from 2.4 to 19.2% by the addition of WAZ and MUAC with WLZ, which captured a wider range of infants in the high-risk category, namely the small and vulnerable [14].

Risk factor analysis found Low Birth weight (LBW) and improper feeding habits to be the main cause of SAM. In our observation, 62.8% of infants with SAM were LBW and that was much more than in the non-SAM group (39.3, p < 0.001) (Table 4 - see PDF). This close correlation supports the conclusions made by Sahu *et al.* in Vellore, India, that showed that LBW (Adjusted Odds Ratio 8.95) and maternal underweight (AOR 6.87) are the most significant predictors of malnutrition, which underscores the intergenerational aspect of the condition [15]. Moreover, even though 65.7% of SAM newborns in our cohort were said to be exclusively breastfed, they managed to present with severe wasting (Table 5 - see PDF). The same "breastfeeding paradox" was also pointed out by Patwari *et al.* who reported that 29 percent of exclusively breastfed children in their study were wasted [11]. This indicates that breastfeeding status is not a sufficient proxy of nutritional safety with regards to maternal malnutrition and possibly suboptimal lactation. Moreover, erroneous feeding routines such as pre-lacteal feeding were remarkably related to SAM (p = 0.024). According to Gashaw *et al.* the colostrum avoidance is still common (28.3) in such environments as Ethiopia, due to the cultural policies that postpone the initiation of breastfeeding and increase the risk of premature growth retardation [16]. Similarly, Negesse *et al.* found of the total infants under six months with various forms of malnutrition, nearly half and a greater proportion of those who were non-exclusively breastfed (NEBF), suffered from malnutrition, including 65 (12.3%) with wasting, 77 (14.4%) underweight and 32 (6.2%) withered [14]. These comorbidities tend to progress into serious conditions; Kassaw *et al.* found that infants with SAM also tend to manifest with acute gastroenteritis (44.9%), sepsis, which complicates clinical management and requires the use of the opportunity of integrated care pathways [17].

## Conclusion:

This study found 23.8% SAM prevalence in infants under six months, with MUAC <11 cm demonstrating balanced sensitivity (69.23%) and specificity (73.09%) for SAM detection. We recommend integrating MUAC as a practical community-based screening tool alongside weight-for- length measurements in infants under six months, extending current protocols used for the 6-59 month age group to enable early identification in resource-limited settings.
